# The Transpolar Drift conveys methane from the Siberian Shelf to the central Arctic Ocean

**DOI:** 10.1038/s41598-018-22801-z

**Published:** 2018-03-14

**Authors:** E. Damm, D. Bauch, T. Krumpen, B. Rabe, M. Korhonen, E. Vinogradova, C. Uhlig

**Affiliations:** 1 Alfred Wegener Institute Helmholtz-Centre for Polar and Marine Research P.O. Box 12061, 27515 Bremerhaven, Germany; 2 GEOMAR Helmholtz-Centre for Ocean Research, Wischhofstrasse1-3, 24148 Kiel, Germany; 3 Finnish Meteorological Institute, Erik Palmenin aukio 1 P.O. Box 503, FI-00101 Helsinki, Finland; 4 Shirshov Institute of Oceanology, Russian Academy of Science, 36, Nahimovskiy prospekt, 117997 Moscow, Russia

## Abstract

Methane sources and sinks in the Arctic are poorly quantified. In particular, methane emissions from the Arctic Ocean and the potential sink capacity are still under debate. In this context sea ice impact on and the intense cycling of methane between sea ice and Polar surface water (PSW) becomes pivotal. We report on methane super- and under-saturation in PSW in the Eurasian Basin (EB), strongly linked to sea ice-ocean interactions. In the southern EB under-saturation in PSW is caused by both inflow of warm Atlantic water and short-time contact with sea ice. By comparison in the northern EB long-time sea ice-PSW contact triggered by freezing and melting events induces a methane excess. We reveal the Ttranspolar Drift Stream as crucial for methane transport and show that inter-annual shifts in sea ice drift patterns generate inter-annually patchy methane excess in PSW. Using backward trajectories combined with δ^18^O signatures of sea ice cores we determine the sea ice source regions to be in the Laptev Sea Polynyas and the off shelf regime in 2011 and 2015, respectively. We denote the Transpolar Drift regime as decisive for the fate of methane released on the Siberian shelves.

## Introduction

Arctic methane sources are considered to contribute to Arctic amplification of global warming as significant methane emissions into the atmosphere may generate positive feedbacks to global warming^1^. However, estimations of methane emissions reveal still large disparities in the Arctic methane budget: bottom-up estimations of all methane emissions are larger than the top-down atmospheric inversions, based on methane atmospheric observations^2^.

Emissions estimated from marine sources, mainly localized on the shallow shelves^3^, are mostly based on sea-air flux calculations during ice-free conditions^4,5^ and ref. therein. Using this method, large uncertainties in estimations might result from detecting source-released methane in sea water which does not finally transit the sea-air interface on the shelves. Instead of efflux on shelves, methane might be trapped in sea ice or in dense shelf water formed during ice formation and subsequently transported by shelf outflow towards the interior Arctic Ocean. Although these pathways are not well constrained, a positive relation between increasing emissions and sea ice decline is reported as feedback to the Arctic amplification of global warming^6^.

There is growing evidence that sea ice is crucial to Arctic methane cycling: atmospheric concentrations are higher over open leads^7^, methane is over-saturated beneath multi-year sea ice^8^ and observed to be taken up in fast ice by freezing^9^. In particular, the mismatch between annual sea-air flux estimates and below-sea ice concentrations in Siberian shelf seas on the one hand^5^ and methane release from sea ice into PSW in the interior Arctic on the other^10^ strongly point to unconsidered shelf-ocean interactions.

As both, the methane uptake- and methane release-locations, are linked by drifting sea ice we hypothesize a “conveyer belt” for transport of Siberian shelf-sourced methane through the Eurasian Arctic within the large Transpolar Drift Stream (TDS). The TDS is one of the two large systems of wind driven sea ice drift and near-surface currents in the Arctic Ocean transporting sea ice from the Siberian coast and shelf across the Arctic Basin into the Fram Strait^11^. Unlike the Beaufort Gyre, that may keep sea ice for several years, the TDS transports mainly first or second year sea ice (FYI & SYI). To test our hypothesis we combined oceanographic and geochemical data sets in the Eurasian Arctic with trajectories of sea ice drift. We show that sea-ice drift pattern impacts methane cycling: Certain regions of sea-ice formation on the shelf seas and the drift duration are crucial for the amount of shelf-sourced methane finally released. This affects the extent of methane super-saturation in PSW.

## Results

### Methane inventory in Polar surface water (PSW)

**Figure 1:**
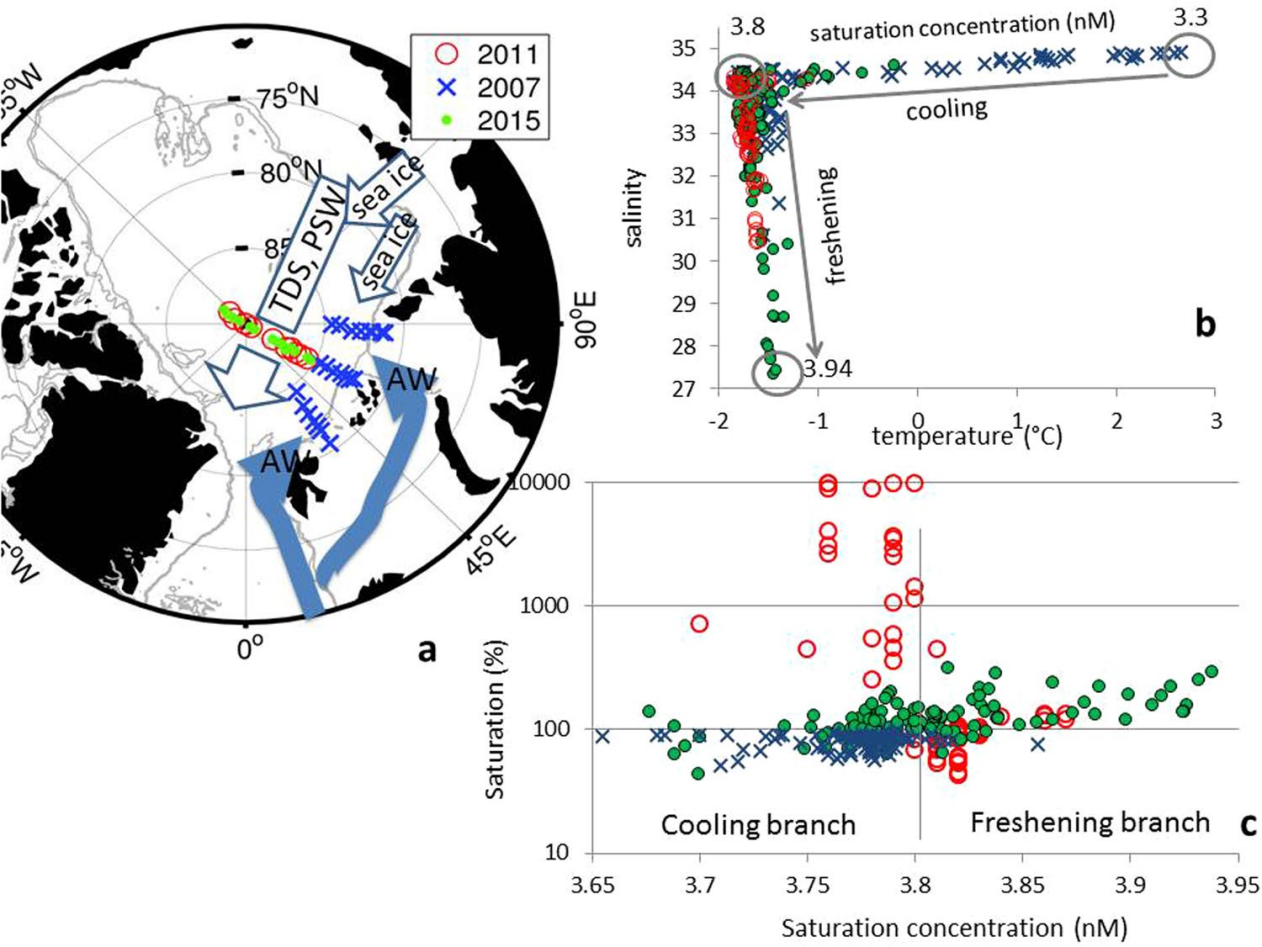
(**a**) Transpolar Drift Stream (TDS), Polar surface water (PSW) and Atlantic water (AW) circulation pattern in the Eurasian Basin (EB). (**b**) Temperature vs. salinity in PSW; grey arrows show increasing saturation concentration of methane (calculated). (**c**) Methane saturation concentration vs. methane saturation separated in a cooling and freshening branches. (**a**) The PSW in the southern EB is influenced by inflowing AW (blue arrows) from SW and by the TDS (white arrows) flowing in the opposite direction i.e. from NE. (**b**) Cooling-down is evident in the AW-influenced PSW (blue crosses) in the southern EB. In comparison, freshening by sea ice melt is most apparent in TDS-influenced PSW in the northern EB (green dots and red circles). Two separate branches of increasing methane saturation concentrations are related to cooling and freshening (grey arrows). (**c**) Saturation concentration vs. saturation reveals methane under-saturated to slightly super-saturated PSW in the southern EB along the cooling branch (blue crosses) and large differences in supersaturation from the cooling to the freshening branch in the northern EB between 2011 (red circles) and 2015 (green dots). Map and plots are generated with MATLAB 2013b.

We detected different levels of methane saturation related to the atmospheric equilibrium in ice covered Polar surface water (PSW) in the Eurasian Basin (EB) (Fig. 1). PSW comprises all water within the winter mixed-layer i.e. the seasonal meltwater layer on top and the remainder of the water from winter mixing beneath^12,13^.

The methane saturations were calculated by applying the equilibrium concentration of methane in sea water with the atmosphere as function of temperature and salinity^14^ and using an atmospheric mole fraction of 1.88, the monthly mean from August 2007, 2011 and 2015 (NOAA global sampling networks, sampling station Zeppelin station, Spitsbergen, http://www.esrl.noaa.gov).

The methane saturation concentration in northern North Atlantic amounts 2.7 nM, i.e. sea water with temperature of 10 °C and a salinity of 35^15^. When entering the Arctic Ocean the sea water temperature drops to less than 3 °C (salinity 34.9) which corresponds to a nearly 20% enhanced solubility capacity, i.e. a saturation concentration of 3.3 nM. On top, melted ice reduces the salinity to 34.3 while the temperature drops to the freezing point resulting in a saturation concentration of 3.8 nM which finally accounts for an enhanced solubility capacity of nearly 30%. This saturation concentration designates the highest saturation concentration reachable by cooling, which means that this value (3.8 nM) also limits the range where the methane saturation level is mainly cooling-triggered.

When achieved, further enhancements in saturation concentrations are caused by freshening during sea ice melt or fresh water inflow, i.e. in that range the methane saturation level is freshening-triggered. We calculated the highest equilibrium concentration of 3.94 nM when salinity drops to 27.3.

Summarized, the methane saturation concentration calculated as a function of sea water temperature and salinity allows separating the increasing saturation capacity in ice covered sea water to a mainly cooling-induced and a mainly freshening-induced branch (Fig. 1). The cooling branch includes two types of cooling, i.e. cooling of inflowing Atlantic water and cooling by sea ice coverage. The freshening branch points to a decrease in salinity by sea ice melt and fresh water inflow. Our data show spatial (between southern and northern EB) and temporal (northern EB in 2011 and 2015) differences in methane saturation related to both branches (Fig. 1).

### AW-influenced PSW in the southern EB

**Figure 2:**
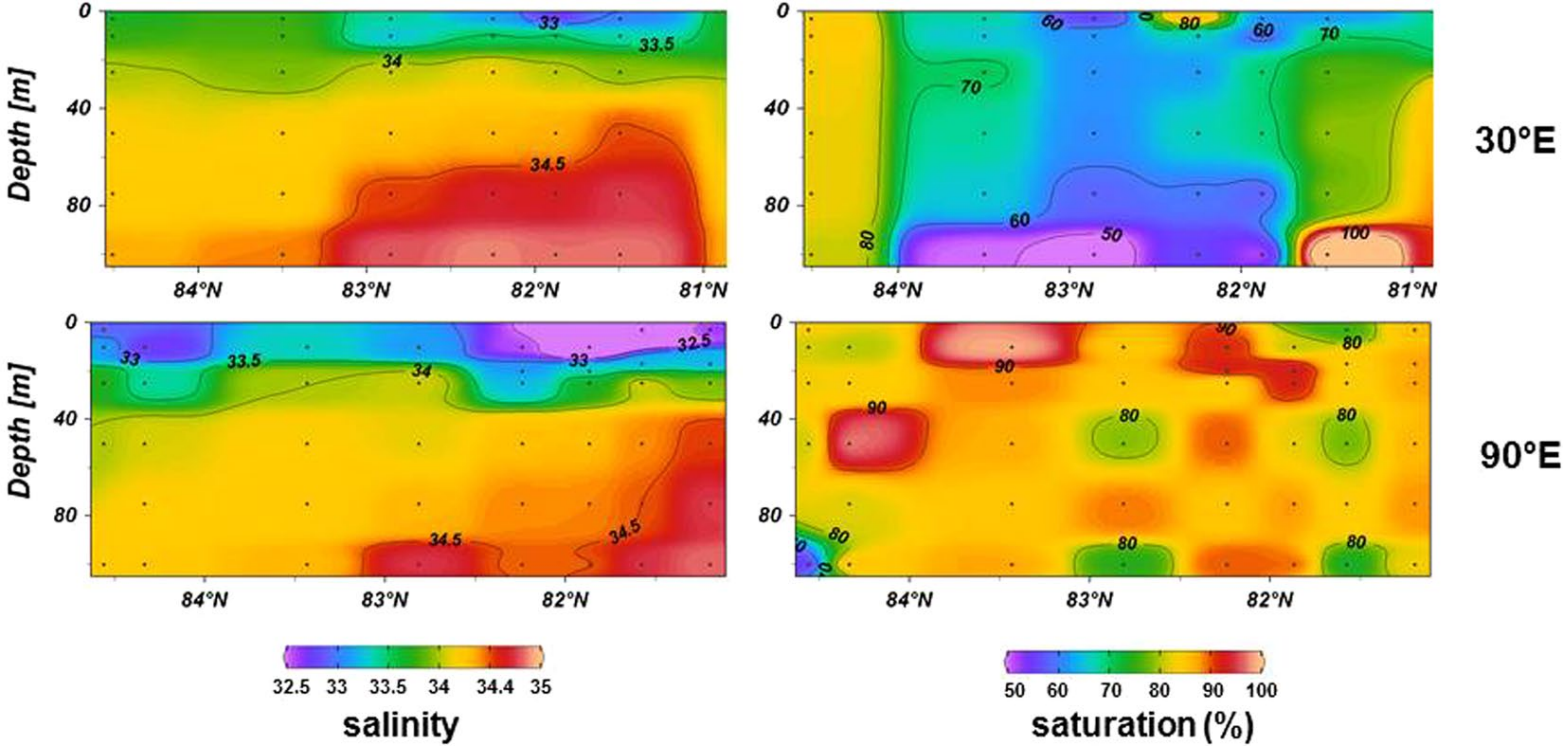
Salinity and methane saturation along the 30°E and 90°E section in the southern Eurasian Basin in 2007. The core of Atlantic water is mainly methane under-saturated along the 30°E. Enhanced methane saturation at the most southern stations is induced by the shelf water outflow from the Barents Sea shelf. The most northern station is less influenced by Atlantic water inflow. Further east, at the 90°E transect methane saturation is increasing and more homogeneously.

In the southern EB Atlantic water (AW) enters the Arctic Ocean. Coming from the south, inflowing water is cooled down by ~10 °C to near-freezing temperatures during the journey through the Nordic seas and the Fram Strait^15^. Methane is mainly under- to slightly super-saturated and mostly localized along the cooling branch (Fig. 1). This pattern reflects both the impact of cooled down AW and methane release from sea ice. Under-saturation is most evident at the westernmost section along the 30° E longitude, lessens from west to east and reaches more than 90% at the 90°E section (Fig. 2).

Towards the sea ice covered surface water the under-saturation is decreasing although the saturation concentration is increasing by cooling and freshening. As sea water which is covered for a longer time by sea ice is less under-saturated, methane release from melting sea ice is most likely to reduce the under-saturation (Fig. 2). Although air-sea flux over open leads is also likely to increase the saturation this contribution is expected to be small as in contrast sea ice coverage hampers the diffusive gas exchange^16^ and in 2007 thick sea ice covered the southern EB.

However, the effect of sea ice-released methane to enhance the saturation level remains small in this region as the opposite direction of the ocean current along the Svalbard and Barents Sea continental margin (from south-west)^17^ to the wind driven sea ice drift (from north-east)^18^ results in just a short contact of sea water with the sea ice cover on top. In this regard, the “non-common history” of sea ice and PSW advected along the southern EB focuses the view to the duration of sea water-sea ice contact for the level of saturation generated in PSW.

### TDS-influenced PSW in the northern EB

**Figure 3:**
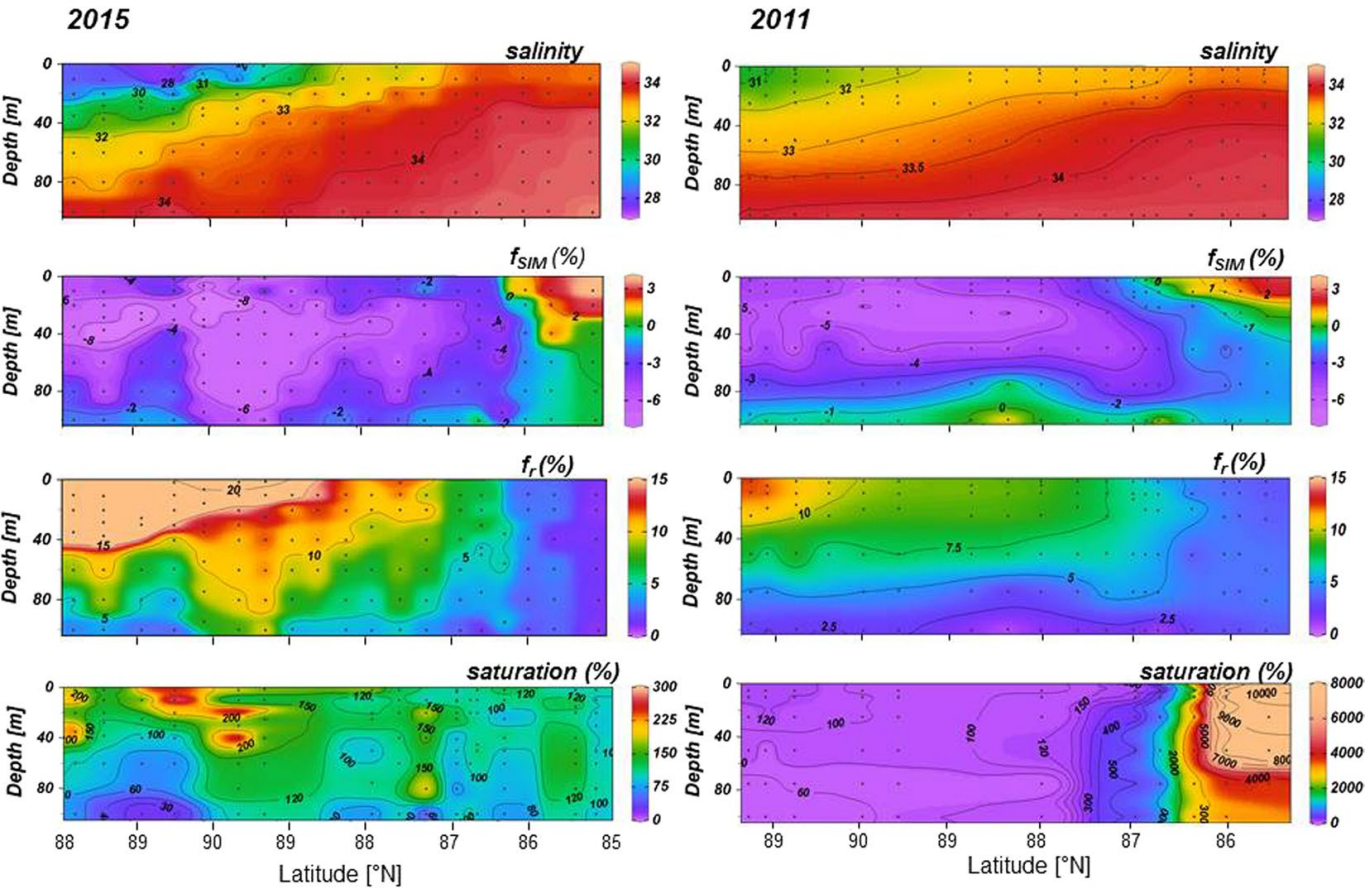
Salinity, fractions of freshwater components, i.e. sea-ice meltwater (f_SIM_) and river waters (fr) and methane saturation (top down) along the 60°E section (from north to south, section location see Fig. 1a) in 2015 (left) and 2011 (right). Fractions of freshwater components are based on mass balance calculations of salinity, nutrients and the stable oxygen isotope composition (δ^18^O) in the water column. The pronounced negative fractions of sea ice meltwater (f_SIM_) shows sea ice production with subsequent brine release, i.e. freezing, as the main process forming PSW in both years. Differences between both years are apparent at the southernmost part of the section. In 2011 negative f_SIM_ cover the whole section with positive values just in the upper ~20 m showing the effect of melting. In 2015 positive (f_SIM_) values are found in the upper ~40 m in that region whereas below 40 m (f_SIM_) values close to zero reflect the negligible sea ice influence on inflowing AW. Fractions of meteoric waters (f_r_) primarily reflect the influence of river waters released to the Siberian shelves and transported via the TDS. The influence of river water is mostly limited to the northern part of the section and is much smaller in 2011 compared to 2015. Whereas the huge methane super-saturation in 2011 is unaffected by river water, the moderate super-saturation in 2015 may have contributions from several sources, i.e. freezing, melting and methane from river water discharge.

The regional contrast to the northern EB, where the PSW remains uninfluenced by AW inflow, is remarkable. We detected in two different years various levels in methane supersaturation ranging from the cooling to the freshening branch (Figs 1 and 3). In 2011 a huge supersaturation is mainly coupled to the cooling branch. However, in contrast to the southern EB where mainly AW inflow generates the cooling, cooling in the northern EB just occurs during ice formation in autumn and winter. In that region the TDS transports about 3.48 × 10^5^ km^2^ of sea ice per winter from the Laptev Sea towards the Fram Strait^19,20^. Both, the wind-driven sea ice drift and near-surface currents are thought to move in the same direction, i.e. from northeast to southwest^21^. During that common journey brine release and haline convection induced by freezing and melting create a strong sea ice-ocean coupling^12,13,22^. Hence the spatial coherence creates a first-year to multiyear sea ice-water contact resulting in a “common history” of sea ice and PSW underneath. In that context methane surplus in PSW clearly appears to be sea-ice-sourced and freezing events as most important for methane release. By comparison, in 2015 we detected just a moderate super-saturation which spreads from the cooling to the freshening branch, i.e. sea ice-released methane is added during freezing and melt events. The different scales of super saturation between 2011 and 2015 at the 60°E transect reveal pronounced inter-annual heterogeneities in methane excess. These perplexing circumstances point to a strong impact of the sea ice type and the duration of sea ice coverage on methane super-saturation in PSW. The variations might be: (I) source –triggered i.e. different sea ice types with different methane amounts incorporated therein, (II) related to varying amounts of methane finally released from sea ice into the PSW and (III) coupled to differences in preservation of methane excess over a time period of several seasons to a year in PSW.

## Discussion

**Figure 4:**
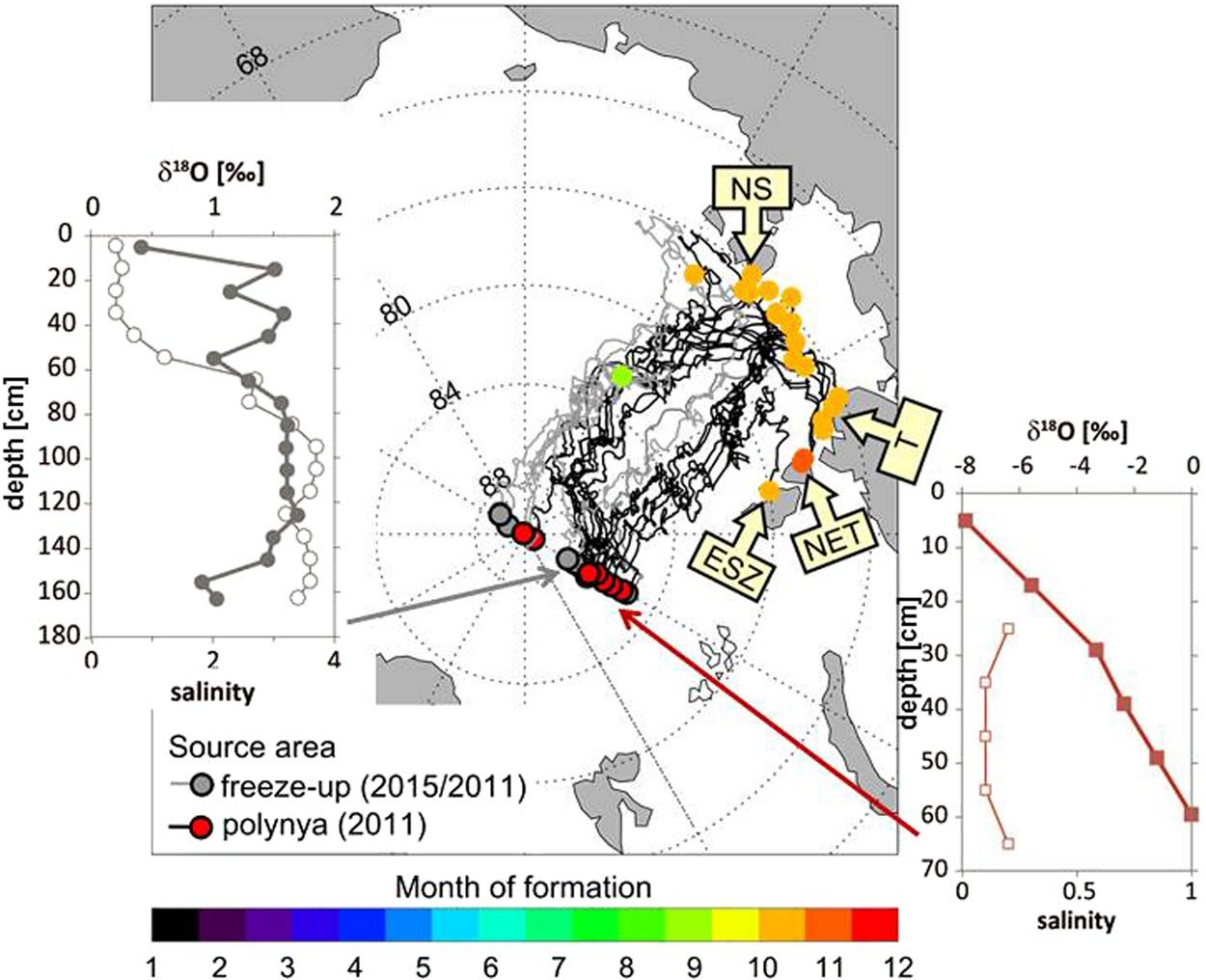
Sea ice drift trajectories leading to the 60°E section and δ^18^O isotopic composition (filled symbols) and salinity (open symbols) in sea ice at this section. Backward drift trajectories from the 60°E section show the sea ice formation areas, i.e. off shore within the Laptev Sea and in the coastal polynya areas. Trajectories were calculated based on a combination of sea ice motion and concentration products from passive microwave satellite data. The colour of the end node indicates the source area of sampled sea ice. Trajectories with red end nodes were formed in polynyas, namely the New Siberian (NS) Polynya, Taymyr (T) Polynya, Northeastern Taymyr (NET) Polynya and East Severnaya Zemlya (ESZ) Polynya. Grey end nodes refer to trajectories that were formed during freeze-up further offshore. The colour coding of the start node characterizes the month of formation (primarily October) of the individual trajectories. The δ^18^O ice isotopic composition reflects the δ^18^O composition of the water column from which each segment of the ice core was formed. Light values below about −4‰ indicate formation in coastal polynyas while values above −2‰ indicate freeze-up formation offshore. Salinity of the ice cores is in all cases below 4. The map is generated with IDL (Interactive Data Langue), software for analysis and visualization of data provided by Harris Geospatial Solutions (http://www.harrisgeospatial.com).
  In 2015 first-year sea ice was trapped at the 60°E section. Except for one station, sea ice originated in the central Laptev Sea where it had been formed 10–11 month earlier during freeze-up (October). After formation, sea ice was advected north by prevailing offshore winds until it crossed the 60°E section. The δ^18^O isotopic composition in sea ice corroborates its formation in the outer shelf regime of the Laptev Sea (Fig. 4). In that region methane concentration with low, nearly background level, values, with small hotspots of methane excess were detected in surface water in summer 2014^5^. When freezing starts in October methane uptake during sea-ice formation is expected to be small. Hence we suggest in 2015 first-year sea ice formed offshore in the Laptev Sea that potentially transported small amounts of methane within the TDS.
  By comparison, 2011 sea ice that crossed the 60°E section was formed 22 month before. In addition to a much slower drift pattern in that year the drift trajectories clearly show that sea ice passing in different segments of the 60°E section was formed in different areas. Sea ice north of 87.5°N was predominantly formed offshore in the Laptev Sea, comparable to sea ice found in 2015. In contrast, sea ice passing between 85° and 87.5°N, i.e. the region with the methane super-saturation up to 10^4^%, was formed in polynya regions near the coast between October and December. The δ^18^O values of this sea ice show significant influence of meteoric water and corroborate sea ice formation in the shallow polynya regions (Fig. 4). Thus this methane-charged sea ice originated in the shallow Laptev Sea Polynyas and was subsequently transported by the TDS to the northern EB in 2011.
  Laptev Sea Polynyas are formed by ice advection away from the coast supplying ice finally transported by the TDS^19^. In the areas of recurring open water new sea-ice formation with brine release over several months creates convection, in shallow areas down to the bottom. As known from a winter study in a polynya region, this convective mixing enhances the turbulence and initiates resuspension of sediments^23^, which eventually favours methane release from the bottom^24^. Once released from sediments, convective mixing also enables rapid transport of methane to the sea surface^24^. We suggest the unique coupling of these processes supports initiating hot spots for methane uptake in sea ice in polynya regions during winter. Beyond the incorporation of methane the uptake of small particles is likely^25^. This could include the transfer of the microbial community as well as organic matter from surface sediments into sea ice during fast freezing events in winter. Hence methane production could occur during sea-ice drift and further enhance methane stocks in sea ice while reduced again by methane oxidation. As both processes are likely and crucial for the dynamic of methane turnover the longer drift duration is essential when sea ice is formed in shallow polynya regions.
(II) A closer look on inter-annual differences in modification of PSW by convective winter mixing point to the freezing process as crucial for methane release from polynya sea ice within the TDS. We observe a relation between the scale of supersaturation and the depth of winter mixing. Winter mixing occurs when sea ice temperature drops and brine release leads to haline convection. The depth of the winter mixed layer indicates to which depth brine release influenced the upper water column during the previous winter^12^. Hence to a certain degree the depth of winter mixing reflects the intensity of winter freezing events. Indeed, the extreme methane super-saturation in 2011 between 85° and 87.5°N was localized in the region with the deepest winter mixed layer depth in that year indicating strong freezing events^26^. This observation is corroborated by the stable oxygen isotope composition (δ^18^O) which shows generally negative fractions of sea-ice meltwater (f_SIM_) in the winter mixed layer and thus reflects the net-influence of sea-ice production with subsequent brine release^27^. Positive sea ice meltwater fractions with f_SIM_ of ~2–3% are available just in the upper ~20 m of the water column in 2011 (Fig. 3). In summary, sea ice that originated in the shallow polynya region and drifts in the TDS discharges shelf-methane into the PSW in the EB during strong freezing events in winter. Although freezing-induced brine release primarily creates the methane excess in PSW, the transport of shelf-methane within sea ice is going on until sea ice is melted. Hence, the extent of sea ice-melt during summer mainly triggers the fate of the residual shelf-methane within sea ice, not released in winter.
(III) In addition, sea-ice melt has various effects on keeping methane excess in PSW over a time period of several seasons: When sea ice starts to melt, methane release from sea ice is ongoing until brine is drained. Hence, during that early stage of sea-ice melt methane excess further increases in PSW. However, during a later stage of sea-ice melt, i.e. when brine drainage ceases but sea ice melt is ongoing, freshwater is added to the PSW and starts to dilute the methane excess in PSW.

(I) We use trajectories from sea ice crossing the 60°E section in 2011 and 2015 to compare drift pattern, age of sea ice and shelf regions of ice formation and find pronounced differences between both years (Fig. 4).

**Figure 5:**
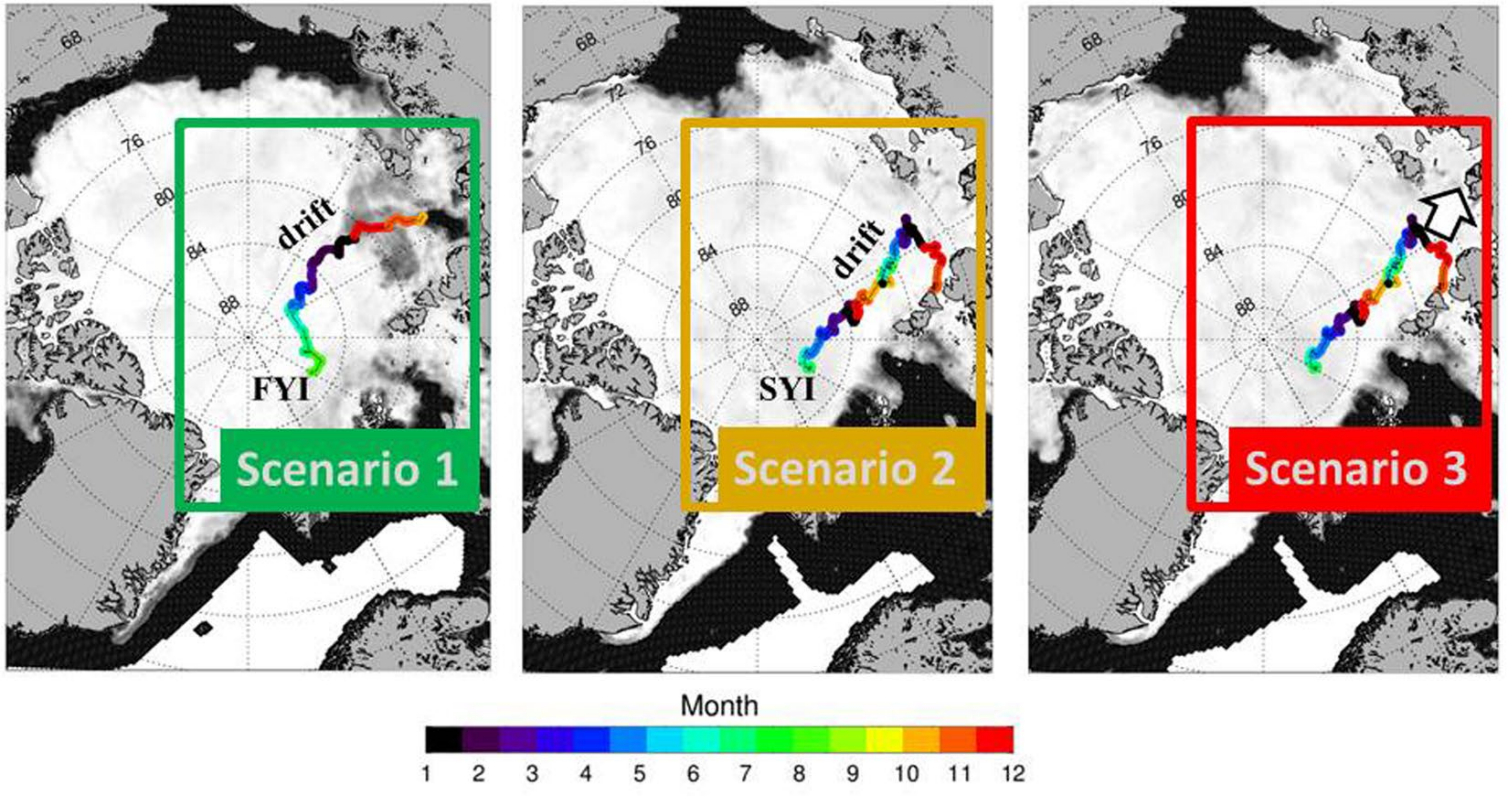
Conceptual scenarios of different sea ice drift patterns decisive for the subsequent methane budget in PSW. Scenario 1 implies: (**a**) Methane is taken up during freeze-up in the interior Laptev Sea from super-saturated surface water. (**b**) Methane charged sea ice starts to drift in the interior Laptev Sea in autumn and reaches the 60°E section in summer, i.e. as first-year sea ice (FYI). Scenario 2 implies: (**a**) Methane is taken up during ice formation in polynya regions in winter from highly super-saturated coastal waters on the Laptev Sea shelf. (**b**) Sea ice starts near the coast in autumn/winter and reaches the 60°E section, after a two year drift i.e. as second-year ice (SYI). Both scenarios imply: Methane discharge during freeze and melt events from drifting sea ice and formation of varying super-saturation in PSW underneath. Scenario 2 may shift to Scenario 3 (hypothetical) when polynya-sea ice becomes disconnected from the TDS (white arrow). Then methane discharge occurs in shelf water when sea ice melts. This finally encourages sea-air flux from the shallow shelves. Abbreviations for polynya names see Fig. 3. The map is generated with IDL (Interactive Data Langue) provided by Harris Geospatial Solutions (http://www.harrisgeospatial.com).

In 2011 the seasonal sea ice melt from the depth of the winter mixed layer was less than 1 m^26^. Thus freshening by sea ice melt and dilution of the methane excess remain small in PSW. Indeed the large methane surplus in 2011 is localized on the cooling branch, i.e. the dilution effect by freshening during sea ice melt remains low. By comparison, in 2015 methane spreads also along the freshening branch, i.e. in that year the melt effect influences the methane excess in PSW. The decreased salinity corroborates the ongoing sea ice melt (Figs 1 and 3). In addition to the dilution effect of sea-ice melt, the thin fresh water layer with the summer sea ice on top inhibits efflux and causes the containment of the methane excess in PSW, where methane oxidation is known to be low^10,28^. Fresh water also induces water stratification which finally decelerates the mixing of the methane excess into deeper water masses. Hence, PSW act at least as a temporal store for methane initially exported from Siberian shelves. We calculated methane budgets stored in PSW under consideration of two different transpolar Drift patterns. Remarkable is that different drift patterns correspond also to different types of sea ice (Fig. 5).

Drift scenario 1 shows transport of freeze up-sea ice formed off shore on the Laptev Sea shelf. Coming that way, methane-discharge from drifting sea ice creates super saturation up to 200% in PSW underneath the TDS. A budget calculation with just an average 150% saturation results in 3.8 Tg methane trapped in the top 30 m of PSW in the Transpolar Drift area (estimated as 4 million km^2^). By comparison, drift scenario 2 reflect transport of methane-charged sea ice from the shallow Laptev Sea Polynya regions. Sea ice taken up in these regions and transported within the transpolar Drift system induces hotspots of methane super-saturation of up to 10^4^% in PSW when methane-release occurs, in particular during freezing events. Extrapolating the hotspot size to an area of 100 km^2^ and the top 60 m 3.4 Tg methane is estimated to be released from sea ice from just a small area. The comparable dimensions of methane budgets trapped in sea ice from large and small areas respectively highlight the significance of sea ice drift patterns for storing methane in PSW. A cascade of feedback processes initiated by trapping methane in sea ice on shelves during sea ice formation and released by freezing and melting events, refers to seasonal methane storage in sea ice. This combines remote sources with the locations where discharge occurs, while both, the atmosphere or the ocean, may act as final sink. Remarkable in this regard is that methane transport within sea ice away from the Laptev Sea shelf coincides with a lower methane surplus in shelf water during the sea ice free season^29^ compared to other Arctic shelf regions^5^.

### Outlook

Sea ice drift is forced by the atmospheric circulation^30^. Hence the impact of the drift pattern on pathways of methane emissions focuses the view to complex interactions and on, yet, unconsidered coast-sea ice-ocean-atmosphere coupling. Consequences of inter-annual variations in drift patterns would become particularly significant if sea ice from the Laptev Sea Polynya regions were disconnected from the large Transpolar Drift Stream. Remaining in the shelf area, a no-drift scenario would initiate a closed shelf cycle, i.e. methane uptake into sea ice during ice formation in autumn and again an *in situ* methane release during sea ice melt in spring. A disconnected shelf-ocean methane transport would fail to include storage of methane in PSW and favour methane escape from the shallow shelves directly to the atmosphere during ice-free seasons. This efflux is expected to be in the range of efflux estimated from ocean/rivers and gas hydrates^1^.

The transport of methane from the Arctic shelf regions in sea ice and in surface water is likely the dominant export pathway as shelf water from the Siberian shelves feed mostly the surface layer of the Arctic halocline^27^ and only small contribution of shelf waters formed offshore is found within or below the pycnocline^31^. However an assessment of the portion of methane which is transported from the shelves below the pycnocline needs also to be estimated in future studies.

Our study is focused on sea ice-ocean interaction, while the role of sea ice–air fluxes and oxidation as pathways of methane in the Arctic need further investigation.

Our study confirms that methane release from sea ice is coupled to the ice freeze and melt cycle. Hence the intensity of freeze events in winter and the amount of summer sea ice retreat primarily triggers how much methane is released during transport within the TDS in the central Arctic.

To which extent the interior Arctic Ocean might act as a final or just a temporal sink, i.e. with final efflux to the atmosphere, is another open question. Furthermore, sea ice retreat, thinning, and decreasing multiyear and increasing first-year sea ice will have, yet, unconsidered consequences for the sea ice-air exchange and the source-sink balance of the greenhouse gas methane in the Arctic. In addition to the potential source capacity for efflux from the northern Eurasian Basin, the potential sink capacity of the southern EB for atmospheric methane might be enhanced if the volume of inflowing AW increases and the region becomes seasonally ice free in the future.

The inclusion of methane from super-saturated shelf water or direct sediment discharge into sea ice during ice formation in autumn and winter is a missing link in the Arctic methane cycle. Further elucidation is required by detailed process studies on the shelves.

## Methods

Sampling occurred along the hydrographic transects in the central Arctic Ocean occupied by RV Polarstern in 2007, 2011 and 2015. In August 2007 three transects samples were taken along 30°E, 60°E and 90°E. In August 2011 and in August to September 2015 we sampled along 60°E.

Profiles of salinity and temperature in the water column were obtained using a conductivity–temperature–depth (CTD) system with a Carousel Water Sampler (Sea-Bird Electronics Inc., Washington, USA). Salinity was calibrated using discrete samples from the rosette, and processed with an on-board Salinometer (OPS, Optimare). Details of the measurements and processing are described in Schauer *et al*.^32–34^.

Water samples for measurements of methane concentration and δ^18^O signature of seawater were collected from up to six different depths during the up cast at each CTD station with 10 L Niskin bottles mounted on a rosette sampler. The intakes for the CTD sensors were mounted at the bottom of the frame to avoid disturbance by the frame reaching the sensors during downcast. The bottles were mounted at a similar level or higher than the sensors. As the bottles sample over a vertical distance of about 1 m (length of the Niskin bottles) the disturbance to the sensors during up-cast and corresponding bottle sensor values is considered negligible.

Sea ice samples for δ^18^O signature were taken from ice cores in 2011 and 2015. Sea ice thickness along sampled transects was ~1.3 m to ~2.3 m. The ice was cut into 5 to 10 cm slices, thawed and the water stored for measurements. All water samples from ice cores and water column were analyzed for the oxygen isotope ratios ^18^O/^16^O at the Stable Isotope Laboratory (Oregon State University, United States). All isotope measurements were performed using the classical CO_2_-water equilibrium method^35^. The overall measurement precision for all δ^18^Oanalysis was ±0.04‰ or better. The ^18^O/^16^O ratio is given n respect to V-SMOW in the δ-notation^36^.

Methane concentrations were analysed within a few hours after sampling. The dissolved gases were extracted from the water by vacuum-ultrasonic treatment^37^. The degassing line consists of a sample bottle mounted inside an ultrasonic bath with removable valve connections to a gas burette and a water reservoir bottle. The top of the burette is equipped with a septum port and a gas sample bulb. A vacuum (<20 mbar) is generated within the degassing line before degassing the sample. The sample bottle is then connected and the dissolved gas is driven out of the sample by ultrasonic energy, alternating (5 s on and 10 s off) for about 5 min. The extracted gas is exposed to atmospheric pressure and the volume reduction is compensated by the water from the reservoir bottle. The gas volume is determined on the burette and the gas can then be taken trough the septum for the GC analyses^37^. Aliquots were measured with the head space method for inter calibration. For this the water was immediately filled from the Niskin bottles into 120 ml serum bottles. Bottles were sealed with gastight butyl rubber stoppers and crimped with Aluminium caps. A 5 ml headspace was created by addition of N_2_ gas. The sample bottles were brought to lab temperature (20 °C) while shaking in a horizontal shaker. A subsample of 150 μl from the headspace gas was then injected into the Gas Chromatograph (GC). In 2015 we mainly used the head space method for determining the methane concentration. In 2011 and 2015 we used the GC Agilent GC7890A and in 2007 the GC Chrompack 9003 each with a flame ionization detector (FID). For gas chromatographic separation we used a packed column (Porapac Q 80/100 mesh). The GC oven was operated isothermally (60 °C) and the FID was held at 200 °C. Two sets of standard gas mixtures were used for calibration. The standard deviation of duplicate analyses was 5%. This overall error is almost exclusively due to the gas extraction procedure, the GC precision had an error of 1%.

The δ^18^O based calculation of water mass fractionation is based on S/δ^18^O mass balances of marine water, meteoric or river water and sea-ice meltwater^27,38^. The calculations used in this manuscript follow the calculations used in Bauch *et al*., 2011 and are briefly summarized here:

Salinity and δ^18^O show a first order linear correlation due to the mixture of ~0‰ δ^18^O marine water with significant amounts of isotopically depleted meteoric water. Meteoric water consists of river runoff and local precipitation, with similar isotopic composition due to their common source and is referred to as river water within this study. The deviations from the linear correlation are caused by sea-ice processes. In the southern Eurasian Basin the contributing water masses are river water, sea-ice meltwater and Atlantic-derived waters that can be separated by simple 3-component mass balance calculations^38,39^. The marine source in the northern and eastern Eurasian Basin and in the North American basins (Makarov and Canadian basins) is expected to be a mixture of Atlantic and Pacific-derived waters^27,38,40–42^ and a 4-component mass balance has to be applied. In this study a 4-component N/P-based mass balance is applied. The mass balance is governed by the following equations:

1 $${f}_{a}+{f}_{p}+{f}_{SIM}+{f}_{r}=1$$

2 $${f}_{a}{S}_{a}+{f}_{p}{S}_{p}+{f}_{SIM}{S}_{SIM}+{f}_{r}{S}_{r}={S}_{meas}$$

3 $${f}_{a}{O}_{a}+{f}_{p}{O}_{p}+{f}_{SIM}{O}_{SIM}+{f}_{r}{O}_{r}={O}_{meas}$$

4 $${f}_{a}{P}_{a}+{f}_{p}{P}_{p}+{f}_{SIM}{P}_{SIM}+{f}_{r}{{P}}_{{r}}={P}_{meas}$$

where *f*_a_ is the fraction of Atlantic water, *f*_p_ the fraction of Pacific-derived water, *f*_*SIM*_ the fraction of sea ice meltwater, and *f*_r_ is the fraction of river water. S, O and P with the corresponding subscript are the endmember values and measured values of salinity, δ^18^O and the phosphate value of each endmember or the measured sample. Fixed phosphate endmember values are assigned to river water and sea-ice meltwater (see Tab. 1). We use the measured NO_x_ concentration of each sample and derive individual phosphate (P) endmembers for the Pacific and Atlantic fractions from the “pure Atlantic water line” defined for our study ([NO_x_] = 16.785 $\ast$ [PO_4_] −1.9126^27^; and the “pure Pacific water line” as defined by^40^ ([NO_x_] = 15.314 $\ast$ [PO_4_] −14.395) for each sample. Due to inaccuracies in end-members and measurements, N/P-based calculations may also produce slightly negative fractions *f*_*p*_ of Pacific-derived waters. These however, remain relatively small also within the Atlantic regime (average *f*_*p*_ are ~−2%, with extreme values up to −10%) and are still within the uncertainty (~10%) of the method^42^.

**Table 1:** Endmember values used for the N/P-based four component mass-balance calculations. Numbers given in parentheses are the estimated uncertainties within the last digit in our knowledge of each endmember value. Analytical errors are all considerably smaller.

Endmember	Salinity	δ^18^O (‰)	PO*-based PO* (μmol**∙**kg^−1^)	**N/P-based** PO_4_ (μmol**∙**kg^−1^)
Atlantic Water (f_a_)	34.92(5)	0.3(1)	0.70(5)	0.0596 $\ast$ [NO_x_] + 0.1139 ± 0.02
river water (f_r_)	0	−20(1)	0.1(1)	0.1(1)
sea ice meltwater (f_SIM_)	4(1)	−2(1)	0.4(1)	0.4(1)
Pacific water (f_p_)	32.7(2)	−1.1(2)	2.4(3)	0.0653 $\ast$ [NO_x_] + 0.9400 ± 0.02

A negative sea-ice meltwater fraction *f*_*SIM*_ reflects the amount of water removed by sea-ice formation, and the absolute value is proportional to the subsequent addition of brines to the remaining water. Therefore we refer to negative fractions of sea-ice meltwater also as sea-ice derived brine influence or just brine influence. The sea-ice meltwater fraction does not include meltwater from ice formed from river water; this is river water previously transported by ice and is identified by its δ^18^O and salinity signature, and it is accounted for in *f*_*r*_ accordingly. All fractions are net values reconstructed from the δ^18^O, salinity and the nutrient signature of each sample and are the result of time integrated effects on the sample volume over the residence time of the water.

Sea ice trajectories were used to follow pathways of sea ice and identify source areas by tracking the sampled ice backward using a combination of ice drift and concentration data obtained from low resolution passive microwave satellites. Ice motion data used in this study are provided by different institutions and have been widely used in sea ice studies^18,20,43^. To track sea ice, two different sets of ice drift products were used: During summer month (June – August), the Polar Pathfinder Sea Ice Motion product provided by the National Snow and Ice Data Center (NSIDC) given on a 25 km grid^44^ was applied. During the rest of the year, tracking is forced with sea ice motion data provided by the Center for Satellite Exploitation and Research (CERSAT) at the Institute Francais de Recherche pour d’Exploitation de la Mer (IFREMER). Motion data are available with a grid size of 62.5 km, using time intervals of 3 days for the period between September and May^45^. Sea ice concentration data used in this study are obtained from the NSIDC. Following Krumpen *et al*.^20^ the tracking procedure works as follows: Using motion and concentration data, a specific ice area is tracked backwards until: (a) the ice reaches a position next to a coastline, (b) the ice concentration at a specific location reaches a threshold value of >15% when ice parcels are considered lost, or (c) the tracking time exceeds 4 years.

### Data availability

Data available from Pangaea repository at https://www.pangaea.de/?t=Oceans&q=Bauch%2C+Dorothea.
